# Assessing the empirical validity of alternative multi-attribute utility measures in the maternity context

**DOI:** 10.1186/1477-7525-7-40

**Published:** 2009-05-06

**Authors:** Stavros Petrou, Jane Morrell, Helen Spiby

**Affiliations:** 1 National Perinatal Epidemiology Unit, Department of Public Health, University of Oxford (Old Road Campus), Oxford, UK; 2 Health Economics Research Centre, Department of Public Health, University of Oxford (Old Road Campus), Oxford, UK; 3 Centre for Health and Social Care Research, School of Human and Health Sciences, University of Huddersfield, Huddersfield, UK; 4 Mother and Infant Research Unit, Department of Health Sciences, University of York, York, UK

## Abstract

**Background:**

Multi-attribute utility measures are preference-based health-related quality of life measures that have been developed to inform economic evaluations of health care interventions. The objective of this study was to compare the empirical validity of two multi-attribute utility measures (EQ-5D and SF-6D) based on hypothetical preferences in a large maternity population in England.

**Methods:**

Women who participated in a randomised controlled trial of additional postnatal support provided by trained community support workers represented the study population for this investigation. The women were asked to complete the EQ-5D descriptive system (which defines health-related quality of life in terms of five dimensions: mobility, self care, usual activities, pain/discomfort and anxiety/depression) and the SF-36 (which defines health-related quality of life, using 36 items, across eight dimensions: physical functioning, role limitations (physical), social functioning, bodily pain, general health, mental health, vitality and role limitations (emotional)) at six months postpartum. Their responses were converted into utility scores using the York A1 tariff set and the SF-6D utility algorithm, respectively. One-way analysis of variance was used to test the hypothetically-constructed preference rule that each set of utility scores differs significantly by self-reported health status (categorised as excellent, very good, good, fair or poor). The degree to which EQ-5D and SF-6D utility scores reflected alternative dichotomous configurations of self-reported health status and the Edinburgh Postnatal Depression Scale score was tested using the relative efficiency statistic and receiver operating characteristic (ROC) curves.

**Results:**

The mean utility score for the EQ-5D was 0.861 (95% CI: 0.844, 0.877), whilst the mean utility score for the SF-6D was 0.809 (95% CI: 0.796, 0.822), representing a mean difference in utility score of 0.052 (95% CI: 0.040, 0.064; *p *< 0.001). Both measures demonstrated statistically significant differences between subjects who described their health status as excellent, very good, good, fair or poor (*p *< 0.001), as well as monotonically decreasing utility scores (test for linear trend: *p *< 0.001). The SF-6D was between 29.1% and 423.6% more efficient than the EQ-5D at detecting differences in self-reported health status, and between 129.8% and 161.7% more efficient at detecting differences in the Edinburgh Postnatal Depression Scale score. In addition, the SF-6D generated higher area under the curve (AUC) scores generated by the ROC curves than the EQ-5D, indicating greater discriminatory power, although in all but one analysis the differences in AUC scores between the measures were not statistically significant.

**Conclusion:**

This study provides evidence that the SF-6D is an empirically valid and efficient alternative multi-attribute utility measure to the EQ-5D, and is capable of discriminating between external indicators of maternal health.

## Background

Economic evaluations of health care interventions are increasingly being conducted throughout the industrialised world to inform the efficient allocation of finite health care resources [1]. In many jurisdictions, cost-utility analysis represents the preferred technique of economic evaluation [2]. The technique allows health interventions, within and across health care programmes, to be compared in terms of their costs and the health improvements they procure, thereby permitting finite health care resources to be allocated on a utilitarian 'cost per unit of health improvement' basis [3]. Potential measures for estimating health improvements within a cost-utility framework include the quality-adjusted life year (QALY) [4], the healthy years equivalent (HYE) [5] and the saved young life equivalent (SAVE) [6]. The QALY synthesises information on the length of life and the health-related quality of life into a single measure of health outcome, and is the most widely used of the various measures.

Alternative approaches to deriving the health-related quality of life component of the QALY for the purposes of economic evaluation include scaling techniques, such as the standard gamble, time trade-off and person trade-off approaches, and multi-attribute utility measures, which are essentially health status classification systems with pre-existing utility scores (or preference weights) that can be attached to each permutation of responses [7]. In practice, multi-attribute utility measures have acted as the primary source of data for QALY estimation in cost-utility analyses [8,9]. The available multi-attribute utility measures include the Quality of Well-Being Scale [10], Rosser-Kind Classification of Illness States [11], Health Utilities Index (HUI) [12], EQ-5D [13], 16D [14], 17D [15], Assessment of Quality of Life instrument (AQoL) [16] and SF-6D [17]. The EQ-5D and the HUI are currently the most widely used of the multi-attribute utility measures. However, the recent development of the SF-6D, which is derived from the Short Form 36 (SF-36) health survey [18], one of the most widely used generic measures of health-related quality of life in health services research, has the potential to considerably increase the derivation of QALY estimates using existing and future data sets [17].

The selection of a multi-attribute utility measure for application within an economic evaluation framework should be informed by its psychometric properties in each clinical context, including its practicality, reliability and validity [19]. A crucial requirement for health economists is that there is evidence for the measure's *empirical validity*, that is, that the measure generates utility scores (essentially the health-related quality of life component of the QALY) that reflect people's preferences. Brazier and Deverill [4] propose a hierarchy of evidence for establishing the empirical validity of multi-attribute utility measures: revealed preference data (i.e. preferences revealed from actual decisions), stated preference data (i.e. expressed preferences using techniques such as contingent valuation [7]), and hypothetical preferences (i.e. preferences that are hypothesised or constructed by researchers) [19]. Most commonly, establishing the empirical validity of multi-attribute utility measures has involved examining whether the utility scores generated by the measures reproduce hypothesised differences between groups of individuals or patients [19]. To our knowledge, no study to date has assessed the empirical validity of multi-attribute utility measures in the maternity context. The objective of this study was to compare the empirical validity of EQ-5D versus SF-6D utility scores based on hypothetical preferences in a large maternity population in England. In so doing, we aim to provide evidence on the relative merits of two prominent multi-attribute utility measures for those involved in analysing and interpreting economic evaluations in the maternity context.

## Methods

### Study population

Women who participated in a randomised controlled trial of additional postnatal support provided by trained community support workers represented the study population for this investigation [20,21]. The trial recruited 623 English-speaking women from a university teaching hospital in Sheffield, northern England, over the period October 1996 to November 1997. Women were eligible for the trial if they were aged 17 years or over and had given birth to a live baby. The population living in the catchment areas of the teaching hospital broadly reflected the age and ethnic profile for the general population of England and Wales, but had a slightly lower fertility rate compared with the general population and a higher proportion living in underprivileged areas (highest category of Jarman scores) [20]. Women recruited into the trial were more likely to be older, of white ethnic origin, to have used transcutaneous nerve stimulation during labour and to have had a caesarean section than the 1046 women who declined participation [20]. Individual women were randomly allocated to a control group that was offered usual postnatal care at home by community midwives (n = 312) or an intervention group that was also offered a maximum of 10 visits from specifically trained community postnatal support workers for up to three hours per day in the first 28 postnatal days (n = 311). There were no significant differences between the allocation groups in terms of a range of general health and psychosocial outcome measures at six weeks and six months postpartum [20]. Therefore, outcomes for individual women allocated to either of the two groups were pooled for the purposes of our empirical investigation. Further details about the randomised controlled trial, its methodology, outcome measures and response rates are reported elsewhere [20].

### Indicators of health status

Two key outcome measures completed by the women in postal questionnaires at six months postpartum acted as the external indicators of health status in this current investigation. The first was general health status, which was categorised as excellent, very good, good, fair or poor. Self-reported health status has been shown to have high internal consistency, construct validity and reliability, as well as representing a good predictor of morbidity and mortality [22,23]. The second was the Edinburgh Postnatal Depression Scale (EPDS), a validated and widely used non-diagnostic instrument for indicating a woman's risk of postnatal depression, a distressing mental disorder more prolonged than the blues (which tend to occur in the first week after delivery) but less severe than puerperal psychosis [24].

### Multi-attribute utility measures

As part of the postal questionnaire completed at six months postpartum, women completed the United Kingdom versions of the EQ-5D [13] and SF-36 [18] instruments (Additional file 1).

The EQ-5D was developed by the 'EuroQol Group', a multi-disciplinary group of researchers from seven centres across five countries, which was formed to generate a cardinal preference-based index of health for comparative purposes [25]. The EQ-5D consists of two principal measurement components. The first is a descriptive system which defines health-related quality of life in terms of five dimensions: 'mobility', 'self care', 'usual activities', 'pain/discomfort' and 'anxiety/depression' [13,25]. Responses in each dimension are divided into three ordinal levels, coded: (1) no problems; (2) some or moderate problems; and (3) severe or extreme problems. The second measurement component of the EQ-5D consists of a 20 cm vertical visual analogue scale ranging from 100 (best imaginable health state) to 0 (worst imaginable health state), which provides an indication of the subject's own assessment of their health status on the day of the survey [13,25]. The women in the present study were asked to complete the EQ-5D descriptive system and not the visual analogue scale. The potential responses to the descriptive system can theoretically generate 243 (3^5^) different health states. For the purposes of our investigation, we applied the York A1 tariff to each set of responses to the descriptive system in order to generate an EQ-5D utility score for each woman [26]. The York A1 tariff set had been derived from a survey of the UK population (*n *= 3337), which used the time trade-off valuation method to estimate preference weights for a subset of 45 EQ-5D health states, with the remainder of the EQ-5D health states subsequently valued through the estimation of a multivariate model [26,27]. Utility scores in the York A1 tariff set range from no problems on any of the five dimensions in the EQ-5D descriptive system (value = 1.0) to severe or extreme impairment on all five dimensions (value = -0.594) [27]. The York A1 tariff set represents the recommended general population-based value set for the purposes of economic evaluation in England and Wales [2].

The SF-36 health survey was developed from the RAND Corporation's Health Insurance Experiment in the United States [28]. The SF-36 measures health-related quality of life, using 36 items, across eight dimensions: physical functioning, role limitations (physical), social functioning, bodily pain, general health, mental health, vitality and role limitations (emotional). For each of the eight dimensions, responses to the survey items are transformed onto a 0 to 100 scale, with higher scores indicating higher levels of health-related quality of life. In addition, the SF-36 produces one physical component summary score and one mental component summary score. Although there is extensive evidence demonstrating the ability of the SF-36 dimension and summary scores to describe health differences between patient groups and changes in health over time [29], the scores themselves do not reflect population preferences required for the purposes of QALY estimation. A number of algorithms for deriving health state utility scores from SF-36 responses have been published to date [17,30-33]. For the purposes of this investigation, we applied the SF-6D utility algorithm to each woman's responses to the SF-36 health survey in order to generate a SF-6D utility score for each woman [17]. The SF-6D algorithm reduces the eight dimensions of the SF-36 to six by combining role limitations due to physical and emotional problems and omitting general health perceptions. The combination of levels across the physical functioning, role limitations, social functioning, bodily pain, mental health and vitality dimensions generates 18,000 (6 *x *4 *x *5 *x *6 *x *5 *x *5) unique health states. A study using a fractional factorial design identified 249 health states from this universe of health states, which were valued by a representative sample of 611 members of the UK general population using the standard gamble valuation method [17]. The utility algorithm applied in the present study is based on the econometric model developed by Brazier *et al*. [17] to predict health state valuations for all 18,000 SF-6D health states. The algorithm generates utility scores for health states ranging from no problems on any of the six dimensions in the SF-6D descriptive system (value = 1.0) to the most impaired level on all six dimensions (value = 0.296) [17].

### Statistical methods

All analyses were based upon responses for women who fully completed all external indicators of health status and multi-attribute utility measures at six months postpartum; no replacement or imputation was performed on missing response items. The economic, socio-demographic and clinical characteristics of women who did and did not complete all items for the relevant health measures were compared using the χ^2 ^test.

Descriptive statistics (mean, standard deviation [SD], median, inter-quartile range, minimum, maximum, 95% and 99% confidence intervals [CIs]) for the EQ-5D and SF-6D utility scores were computed. The within-individual difference in mean utility score was tested using the paired *t*-test. The distribution of data points within each SF-6D dimension was calculated in cases where the EQ-5D utility score equalled 1.0 and the SF-6D utility score was less than 1.0. Similarly, the distribution of data points within each EQ-5D dimension was calculated in cases where the SF-6D utility score equalled 1.0 and the EQ-5D utility score was less than 1.0.

The empirical validity of the EQ-5D and SF-6D utility scores was examined in a number of ways. One-way analysis of variance was used to test the hypothetically-constructed preference rule that utility scores should differ significantly between self-reported health status groups and should decrease monotonically with deteriorating self-reported health status [34]. Further, this preference rule was tested for a number of economic, socio-demographic and clinical sub-groups of the study population as previous research had indicated an association between each of these factors and self-reported health status [27].

The ability of the EQ-5D and SF-6D instruments to detect differences in external indicators of health status was tested using the relative efficiency (RE) statistic. Self-reported health status and the EPDS score represented the external indicators of health status in our study. The relative efficiency statistic has been widely applied in the broader health-related quality of life literature [35-40]. It is defined as the ratio of the square of the *t*-statistic of the comparator instrument (assumed to be the SF-6D utility score for the purposes of this study) over the square of the *t*-statistic of the reference instrument (assumed to be the EQ-5D utility score for the purposes of this study) [35]. A relative efficiency score of 1.0 indicates that the SF-6D has the same efficiency as the EQ-5D at detecting differences in external indicators of health status. A value higher than 1.0 indicates that the SF-6D is more efficient than the EQ-5D at detecting differences in external indicators of health status, whilst a score lower than 1.0 indicates that the SF-6D is less efficient than the EQ-5D.

In order to calculate the relative efficiency statistic, self-reported health status had to be converted into a dichotomous variable by dividing the study population into two groups. The cut-off point used to create this dichotomous variable is necessarily arbitrary and may lead to different conclusions depending on which cut-off is chosen [41]. Therefore, self-reported health status was dichotomised in alternative ways: (i) excellent *versus *very good, good, fair or poor, (ii) excellent or very good *versus *good, fair or poor, (iii) excellent, very good or good *versus *fair or poor, and (iv) excellent, very good, good or fair *versus *poor, and the relative efficiency statistic was recalculated. Similarly, two alternative cut-off points were applied to the EPDS score: (i) < 13 *versus *≥ 13, and (ii) < 10 *versus *≥ 10, on the basis that a score of 13 or more is considered to indicate a significant 'case' of postnatal depression, whilst scores of 10 to 12 indicate a borderline 'case' [24]. In addition, because of concerns that the SF-6D utility algorithm might over predict the value of the poorest health states [17], which is reflected by the different lower bounds of the EQ-5D and SF-6D utility scales, all relative efficiency statistics were recalculated for a more restricted sample of women for whom both EQ-5D and SF-6D utility scores were between 0.296 (the lower bound of the SF-6D utility scale) and 1.0.

Finally, the discriminatory properties of the EQ-5D and SF-6D instruments in a maternity context were compared using receiver operating characteristic (ROC) curves [42]. The ROC curve procedure provides a useful method of evaluating the performance of multi-attribute utility measures against external indicators of health status. For the purposes of our analysis, dichotomous variables of self-reported health status and the EPDS score were adopted as the external indicators. The multi-attribute utility measure that generates the largest area under the ROC curve is regarded as the most sensitive at detecting differences in the external indicator. A measure with perfect discrimination would generate an area under the curve (AUC) score of 1.0, whilst a measure with no discriminatory power would generate an AUC score of 0.5.

All *p*-values were considered statistically significant if they were less than 0.05. All analyses were performed with a microcomputer using Statistical Package for the Social Sciences (SPSS) (version 15; SPSS Inc, Chicago, Illinois, USA) software.

## Results

Of the 623 women who participated in the randomised controlled trial of additional postnatal support (20), 493 (79.1%) completed all relevant health measures for the purposes of our investigation. An examination of the characteristics of the women who did not fully complete these measures revealed that they were more likely to be less than 25 years of age, of non-white ethnic origin, unemployed, living in rented accommodation and without a car (*p *< 0.01). A full breakdown of the economic, socio-demographic and clinical characteristics of the study population is available from the authors upon request.

Descriptive statistics of the EQ-5D and SF-6D utility scores are presented in Table 1. The mean utility score for the EQ-5D was 0.861 (95% CI: 0.844, 0.877), whilst the mean utility score for the SF-6D was 0.809 (95% CI: 0.796, 0.822), representing a mean difference in utility score of 0.052 (95% CI: 0.040, 0.064; *p *< 0.001) that exceeded the utility score difference of 0.03 cited as a minimum clinically important difference for evaluative purposes [43,44].

**Table 1:** Descriptive statistics of EQ-5D and SF-6D utility scores (n = 493)

	EQ-5D utility score	SF-6D utility score
Mean	0.861	0.809
Standard deviation	(0.181)	(0.140)
Median	0.848	0.830
Inter-quartile range	(0.796, 1.000)	(0.706, 0.938)
Minimum	-0.077	0.374
Maximum	1.000	1.000
95% CI	(0.844, 0.877)	(0.796, 0.822)
99% CI	(0.838, 0.883)	(0.792, 0.826)
Mean difference (95% CI)	0.052 (0.040, 0.064)*	

CI denotes confidence interval.

* *p*-value < 0.001

A total of 177 women (35.9% of analysed sample) had an EQ-5D utility score of 1.0 and a SF-6D utility score of less than 1.0. Notably, amongst women who did not identify problems in any of the EQ-5D dimensions, 54 (10.9% of analysed sample), 22 (4.5%), 27 (5.5%), 54 (10.9%), 144 (29.2%) and 168 (34.1%) identified problems (levels 2–6) on the physical functioning, role limitations, social functioning, bodily pain, mental health and vitality dimensions of the SF-6D, respectively. In contrast, only 1 woman (0.2% of analysed sample), who identified moderate pain or discomfort on the EQ-5D descriptive system, had a SF-6D utility score of 1.0 and an EQ-5D utility score of less than 1.0.

**Figure 1:**
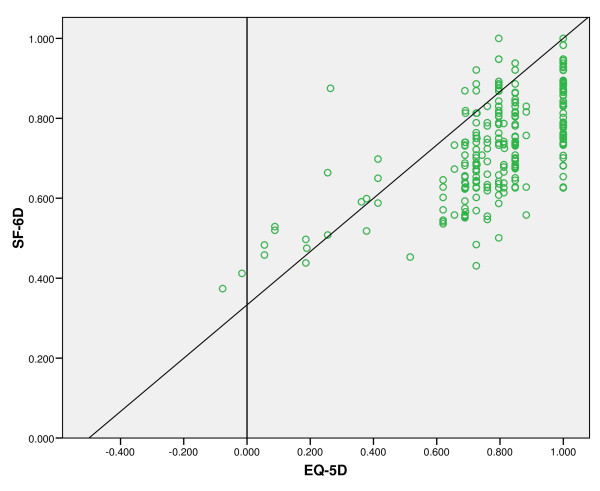
**Scatter plot of paired EQ-5D and SF-6D utility scores**.

Tables 2 and 3, respectively, present mean EQ-5D and SF-6D multi-attribute utility scores for the study population as a whole and for each of the self-reported health status sub-groups. For the study population as a whole, mean EQ-5D and SF-6D multi-attribute utility scores were higher for women of white ethnic origin, women with a car, women living in owner-occupied accommodation, women in paid employment and women who had delivered spontaneously. Both multi-attribute utility measures demonstrated statistically significant differences between women who described their health status as excellent, very good, good, fair or poor (*p *< 0.001). In addition, both multi-attribute utility measures generated utility scores, which decreased monotonically with deteriorating self-reported health status (test for linear trend: *p *< 0.001). The mean EQ-5D utility score was greater than the mean SF-6D utility score for women who described their health status as excellent (0.964 *versus *0.916), very good (0.908 *versus *0.856) or good (0.819 *versus *0.741), but lower for women who described their health status as fair (0.651 *versus *0.652) or poor (0.366 *versus *0.507). This reflected, in part, the distribution of data points illustrated in Figure 1 with the EQ-5D yielding utility scores as low as -0.077, whilst the minimum utility score generated by the SF-6D was much higher up the utility scale (0.374). When the data were analysed within each of the economic, socio-demographic and clinical sub-groups, both multi-attribute utility measures demonstrated statistically significant differences between women who described their health status as excellent, very good, good, fair or poor (*p *< 0.001), as well as monotonically decreasing utility scores (test for linear trend: *p *< 0.001). The only sub-groups for which this was not the case were women of non-white ethnic origin and women who had delivered twins, although this might be explained by the relatively small numbers for these sub-groups (*n *= 27 and *n *= 5, respectively).

**Table 2:** Relationship between mean EQ-5D utility scores and self-reported health status (n = 493)

Group	Overall utility score	Self-reported health status					*p***-**value*
			
		Excellent	Very good	Good	Fair	Poor	
*All women*	0.861	0.964	0.908	0.819	0.651	0.366	<0.001
							
*Age (years)*							
17–24	0.848	0.949	0.887	0.815	0.583	-0.077	<0.001
25–34	0.866	0.973	0.912	0.813	0.672	0.678	<0.001
35–44	0.855	0.954	0.923	0.861	0.659	0.121	<0.001
							
*Ethnicity*							
White	0.864	0.968	0.911	0.821	0.659	0.366	<0.001
Non-white	0.793	0.840	0.835	0.804	0.508	-	0.125
							
*Car ownership*							
Yes	0.876	0.963	0.915	0.825	0.714	0.455	<0.001
No	0.798	0.977	0.875	0.804	0.475	-0.077	<0.001
							
*Housing tenure*							
Owner occupier	0.880	0.964	0.913	0.821	0.723	0.490	<0.001
Rented	0.807	0.965	0.888	0.817	0.565	0.243	<0.001
							
*Paid employment*							
Yes	0.894	0.971	0.912	0.852	0.712	0.678	<0.001
No	0.800	0.941	0.894	0.775	0.626	0.055	<0.001
							
*Plurality*							
Singleton	0.859	0.964	0.907	0.818	0.647	0.366	<0.001
Twin	0.970	1.000	1.000	1.000	0.848	-	-
							
*Spontaneous birth*							
Yes	0.867	0.965	0.907	0.824	0.651	-	<0.001
No	0.847	0.961	0.910	0.809	0.623	0.366	<0.001

* ANOVA.

All tests for linear trend were statistically significant (*p *< 0.05) with the exception of non-white ethnic (*p *= 0.068) and twin (*p *= 0.053) sub-groups.

**Table 3:** Relationship between mean SF-6D utility scores and self-reported health status (n = 493)

Group	Overall utility score	Self-reported health status					*p*-value*
			
		**Excellent**	**Very good**	**Good**	**Fair**	**Poor**	
*All women*	0.809	0.916	0.856	0.741	0.652	0.507	<0.001
							
*Age (years)*							
17–24	0.815	0.916	0.849	0.742	0.656	0.374	<0.001
25–34	0.806	0.911	0.856	0.735	0.655	0.583	<0.001
35–44	0.810	0.954	0.866	0.767	0.631	0.461	<0.001
							
*Ethnicity*							
White	0.816	0.924	0.859	0.750	0.656	0.507	<0.001
Non-white	0.690	0.709	0.790	0.666	0.564	-	0.217
							
*Car ownership*							
Yes	0.817	0.914	0.862	0.734	0.676	0.534	<0.001
No	0.776	0.928	0.828	0.759	0.583	0.374	<0.001
							
*Housing tenure*							
Owner occupier	0.821	0.919	0.860	0.728	0.683	0.553	<0.001
Rented	0.778	0.903	0.837	0.771	0.613	0.461	<0.001
							
*Paid employment*							
Yes	0.831	0.924	0.856	0.758	0.678	0.583	<0.001
No	0.767	0.886	0.852	0.716	0.638	0.432	<0.001
							
*Plurality*							
Singleton	0.809	0.915	0.855	0.741	0.652	0.507	<0.001
Twin	0.822	1.000	0.902	0.681	0.628	-	0.109
							
*Spontaneous birth*							
Yes	0.814	0.915	0.858	0.749	0.641	-	<0.001
No	0.799	0.919	0.852	0.724	0.698	0.507	<0.001

* ANOVA.

All tests for linear trend were statistically significant (*p *< 0.05) with the exception of the non-white ethnic sub-group (*p *= 0.077).

The relative efficiency statistic was used to test how efficient the EQ-5D and SF-6D instruments were in detecting differences in external indicators of health status in this context. When the self-reported health status variable was dichotomised in alternative configurations, the SF-6D was found to be between 29.1% (relative efficiency statistic of 1.291 versus 1.0) and 423.6% more efficient than the EQ-5D at detecting differences in self-reported health status (Table 4). Restricting the analyses to women for whom both the EQ-5D and SF-6D utility scores were between 0.296 (the lower bound of the SF-6D utility scale) and 1.0 reduced the study population by 12. Despite the compression of the utility scale, the SF-6D remained between 24.2% and 143.8% more efficient at detecting differences in self-reported health status in this restricted sample (Table 5). When women were categorised in terms of their risk of postnatal depression, the SF-6D was found to be between 129.8% and 161.7% more efficient than the EQ-5D at detecting differences between alternative EPDS profiles in the complete sample and between 133.0% and 209.6% more efficient in the restricted sample (Table 6).

**Table 4:** Efficiency of multi-attribute utility measures to detect differences in self-reported health status; all women (n = 493)

Measure	Categorisation of self-reported health status	Utility score		*t*-test^a^		Relative efficiency^b^	ROC curve	
				
		Mean	(SD)	*t*-statistic	*p*-value		Area^c^	95% CI
EQ-5D	Excellent	0.964	(0.085)	9.334	<0.001	1.000	0.721*	(0.666, 0.776)
	Very good, good, fair or poor	0.837	(0.189)					
								
SF-6D	Excellent	0.916	(0.091)	10.604	<0.001	1.291	0.798*	(0.748, 0.849)
	Very good, good, fair or poor	0.784	(0.138)					
								
EQ-5D	Excellent or very good	0.925	(0.119)	9.156	<0.001	1.000	0.756*	(0.709, 0.802)
	Good, fair or poor	0.765	(0.213)					
								
SF-6D	Excellent or very good	0.874	(0.108)	14.205	<0.001	2.407	0.841*	(0.804, 0.877)
	Good, fair or poor	0.712	(0.125)					
								
EQ-5D	Excellent, very good or good	0.890	(0.145)	7.222	<0.001	1.000	0.849*	(0.790, 0.908)
	Fair or poor	0.616	(0.258)					
								
SF-6D	Excellent, very good or good	0.830	(0.127)	10.742	<0.001	2.212	0.852*	(0.800, 0.905)
	Fair or poor	0.634	(0.119)					
								
EQ-5D	Excellent, very good, good or fair	0.867	(0.169)	3.469	0.018	1.000	0.814*	(0.633, 0.996)
	Poor	0.366	(0.353)					
								
SF-6D	Excellent, very good, good or fair	0.813	(0.136)	7.938	<0.001	5.236	0.847*	(0.686, 1.000)
	Poor	0.507	(0.093)					

SD denotes standard deviation. ROC denotes receiver operating characteristic. CI denotes confidence interval.

^a ^Not assuming equality of variance as Levene test showed statistically significant differences in variances between self-reported health status groups.

^b ^Relative efficiency statistic is referenced to 1.0 for the EQ-5D measure. A value higher than 1.0 indicates that the SF-6D is more efficient than the EQ-5D in detecting differences between women in terms of their self-reported health status.

^c ^Area under receiver operating characteristic (ROC) curves; * *p *< 0.05 indicates that area under the ROC curve was statistically significantly greater than 0.5 and that measure has discriminatory power.

**Table 5:** Efficiency of multi-attribute utility measures to detect differences in self-reported health status; women for whom both utility scores were between 0.296 and 1.0 (n = 481)

Measure	Categorisation of self-reported health status	Utility score		*t*-test^a^		Relative efficiency^b^	ROC curve	
				
		**Mean**	**(SD)**	***t*-statistic**	***p*-value**		**Area^c^**	**95% CI**
EQ-5D	Excellent	0.964	(0.085)	8.682	<0.001	1.000	0.712*	(0.655, 0.768)
	Very good, good, fair or poor	0.861	(0.137)					
								
SF-6D	Excellent	0.916	(0.091)	10.038	<0.001	1.337	0.792*	(0.741, 0.844)
	Very good, good, fair or poor	0.794	(0.129)					
								
EQ-5D	Excellent or very good	0.931	(0.103)	10.029	<0.001	1.000	0.747*	(0.699, 0.795)
	Good, fair or poor	0.804	(0.143)					
								
SF-6D	Excellent or very good	0.876	(0.106)	13.816	<0.001	1.898	0.835*	(0.797, 0.872)
	Good, fair or poor	0.725	(0.114)					
								
EQ-5D	Excellent, very good or good	0.897	(0.124)	8.672	<0.001	1.000	0.829*	(0.763, 0.896)
	Fair or poor	0.716	(0.126)					
								
SF-6D	Excellent, very good or good	0.833	(0.123)	9.663	<0.001	1.242	0.834*	(0.775, 0.894)
	Fair or poor	0.661	(0.105)					
								
EQ-5D	Excellent, very good, good or fair	0.882	(0.134)	6.482	0.018	1.000	0.720	(0.471, 0.970)
	Poor	0.678	(0.053)					
								
SF-6D	Excellent, very good, good or fair	0.818	(0.l30)	10.121	0.006	2.438	0.770*	(0.544, 0.997)
	Poor	0.583	(0.039)					

SD denotes standard deviation. ROC denotes receiver operating characteristic. CI denotes confidence interval.

^a ^Not assuming equality of variance as Levene test showed statistically significant differences in variances between self-reported health status groups.

^b ^Relative efficiency statistic is referenced to 1.0 for the EQ-5D measure. A value higher than 1.0 indicates that the SF-6D is more efficient than the EQ-5D in detecting differences between women in terms of their self-reported health status.

^c ^Area under receiver operating characteristic (ROC) curves; * *p *< 0.05 indicates that area under the ROC curve was statistically significantly greater than 0.5 and that measure has discriminatory power.

**Table 6:** Efficiency of multi-attribute utility measures to detect differences in postnatal depression

Measure	Categorisation of postnatal depression risk score	Utility score		*t*-test^a^		Relative efficiency^b^	ROC curve	
				
		**Mean**	**(SD)**	***t*-statistic**	***p*-value**		**Area^c^**	**95% CI**
*All women (n = 493)*								
EQ-5D	EPDS score < 13	0.885	(0.147)	4.332	<0.001	1.000	0.696*	(0.615, 0.777)
	EPDS score ≥ 13	0.738	(0.244)					
								
SF-6D	EPDS score < 13	0.830	(0.129)	7.008	<0.001	2.617	0.767*	(0.697, 0.837)
	EPDS score ≥ 13	0.696	(0.132)					
								
EQ-5D	EPDS score < 10	0.896	(0.142)	5.404	<0.001	1.000	0.679*	(0.619, 0.739)
	EPDS score ≥ 10	0.780	(0.208)					
								
SF-6D	EPDS score < 10	0.843	(0.125)	8.192	<0.001	2.298	0.749*	(0.696, 0.802)
	EPDS score ≥ 10	0.724	(0.133)					
								
*Women for whom both utility scores were between 0.296 and 1.0 (n = 481)*								
EQ-5D	EPDS score < 13	0.891	(0.130)	3.699	<0.001	1.000	0.664*	(0.579, 0.750)
	EPDS score ≥ 13	0.806	(0.152)					
								
SF-6D	EPDS score < 13	0.833	(0.126)	6.509	<0.001	3.096	0.760*	(0.685, 0.835)
	EPDS score ≥ 13	0.708	(0.123)					
								
EQ-5D	EPDS score < 10	0.901	(0.128)	5.110	<0.001	1.000	0.662*	(0.600,
	EPDS score ≥ 10	0.821	(0.141)					0.724)
								
SF-6D	EPDS score < 10	0.846	(0.122)	7.800	<0.001	2.330	0.743*	(0.688, 0.798)
	EPDS score ≥ 10	0.735	(0.126)					

EPDS denotes Edinburgh Postnatal Depression Scale. SD denotes standard deviation. ROC denotes receiver operating characteristic. CI denotes confidence interval.

^a ^Not assuming equality of variance as Levene test showed statistically significant differences in variances between self-reported health status groups.

^b ^Relative efficiency statistic is referenced to 1.0 for the EQ-5D measure. A value higher than 1.0 indicates that the SF-6D is more efficient than the EQ-5D in detecting differences between women in terms of their self-reported health status.

^c ^Area under receiver operating characteristic (ROC) curves; * *p *< 0.05 indicates that area under the ROC curve was statistically significantly greater than 0.5 and that measure has discriminatory power.

Finally, the AUC scores generated by the ROC curves provided a further indication of the performance of the two multi-attribute utility measures against external indicators of health status. Both the EQ-5D and SF-6D were able to discriminate between dichotomous configurations of self-reported health status (Tables 4, 5) and dichotomous configurations of risk of postnatal depression (Table 6), (*p *< 0.05). The only exception was the failure of the EQ-5D to discriminate between women who reported excellent, very good, good or fair health and women who reported poor health in the restricted sample (Table 5). In all analyses, the SF-6D generated higher AUC scores than the EQ-5D, indicating greater discriminatory power (Tables 4, 5, 6). However, the corresponding CIs surrounding the AUC scores were only mutually exclusive at the 5% significance level when self-reported health status was dichotomised as excellent or very good *versus *good, fair or poor (Tables 4, 5).

## Discussion

It is now widely accepted that strategies to improve the health and broader well-being of pregnant women and new mothers should be underpinned by a strong evidence base [45,46]. Health economics evidence has made an important contribution towards policy and practice in maternity care in recent years [47]. However, significant gaps remain in our understanding of the production, distribution and evaluation of health and health care for pregnant women and new mothers where health economics evidence could usefully contribute to an efficient and equitable allocation of scarce resources. This is particularly important in the context of childbearing as an event of both health and social importance to women and their families, considerations of investment in maternity service provision and the burden of morbidity for childbearing women [48]. One area where a significant gap in our knowledge remains is an understanding of the relative merits of multi-attribute utility measures that can be incorporated into economic evaluations of maternity care. Although multi-attribute utility measures have been applied in randomised controlled trials of maternity interventions [20,21,49,50], no previous study, to our knowledge, has directly assessed the psychometric properties of these measures in the maternity context. The study reported in this paper investigated the utility scores derived from two multi-attribute utility measures currently in wide use, namely the EQ-5D and SF-6D. As such, it draws upon evidence from a rich data set, namely a randomised controlled trial of additional postnatal support provided by trained community support workers [20].

The main focus of the study centred on the empirical validity of the utility scores generated by the EQ-5D and SF-6D. Given the absence of a manifest gold standard for measuring cardinal preferences for health outcomes, analysts testing the empirical validity of multi-attribute utility measures are required to test whether the utility scores they generate reflect hypothetically-constructed preferences, stated preferences or revealed preferences [34]. The statistical analysis plan adopted by this study focussed on whether the EQ-5D and SF-6D utility scores reflect the hypothetically-constructed preferences of participants in the community postnatal support worker trial. Our prior hypothesis that both the EQ-5D and SF-6D utility scores would differ significantly between self-reported health status groups was met for the study population as a whole, as well as for all but two economic, socio-demographic and clinical sub-groups studied (women of non-white ethnic origin and women who had delivered twins). Our prior hypothesis that both the EQ-5D and SF-6D utility scores would decrease monotonically with deteriorating self-reported health status was also met for the study population as a whole and for all but two of the sub-groups studied. Further, we showed that both measures discriminated between alternative dichotomous configurations of self-reported health status and the EPDS score.

The analytical strategy that we adopted also tested the *degree *to which EQ-5D and SF-6D utility scores reflect external indicators of maternal health. The relative efficiency statistic suggested that the SF-6D was between 29.1% and 423.6% more efficient than the EQ-5D at detecting differences in self-reported health status, and between 129.8% and 161.7% more efficient at detecting differences in the EPDS score. Moreover, the SF-6D remained more efficient at detecting differences in external indicators of maternal health after sensitivity analyses accounted for differences in the standard errors surrounding the two sets of utility scores at the lower end of the utility scale. In addition, the SF-6D generated higher AUC scores than the EQ-5D, indicating greater discriminatory power, although in all but one analysis the differences in AUC scores between the measures were not statistically significant (as indicated by the overlapping 95% CIs).

There are several possible reasons why the SF-6D might be more efficient at detecting external indicators of maternal health than the EQ-5D. First, although both measures are rooted in the World Health Organisation definition of health, which covers physical, mental and social well-being, the SF-6D may tap into broader aspects of health-related quality of life, such as role and social functioning. Second, the SF-6D has a greater number of response items to each of its dimensions, resulting in a larger descriptive system (18,000 health states *versus *243 EQ-5D health states) and, consequently, a possibly greater degree of sensitivity to maternal health indicators. Third, the wording of the SF-6D response items, which includes positive as well as negative aspects of health, might independently result in a greater degree of sensitivity to maternal health indicators. Fourth, the longer time frame covered by the SF-6D, which frames its questions in terms of health 'over the past 4 weeks', as opposed to the time frame covered by the EQ-5D descriptive system, which frames its questions in terms of health 'today', might independently increase its sensitivity to the external indicators of maternal health adopted by this study. Ultimately, a full understanding of the reasons for the greater efficiency of the SF-6D at detecting external indicators of maternal health is beyond the scope of this paper. Separate studies are required to test the hypotheses set out above.

There are a number of caveats to the study results which should be borne in mind by readers. First, the analytical strategy focussed on whether EQ-5D and SF-6D utility scores reflect hypothetically constructed preferences. The external indicator of self-reported health status adopted by our study represents a good predictor of morbidity and mortality [22,23], whilst the external indicator of the EPDS score has been shown to have high sensitivity and specificity against diagnostic criteria in postpartum samples [51]. Ideally, we would also have liked to test the utility scores generated by the EQ-5D and SF-6D measures against stated preferences and revealed preferences. However, stated and revealed preference data were not collected as part of the postnatal support worker trial. Furthermore, markers for revealed preferences such as the purchase of over the counter medications, for which some relevant data were available, are prone to the problem of contaminants and confounding factors, and this would have made it difficult to interpret the basis of those purchasing decisions. Second, all tests of empirical validity that we performed were applied to cross-sectional data collected at six months postpartum. The EQ-5D and SF-6D were not administered at the time of randomisation immediately after delivery and the EQ-5D was not administered at six weeks postnatally (whilst the SF-36 was) and, consequently, we are unable to make any firm assertions about how the EQ-5D might perform longitudinally [52,53]. Third, the women in our study only completed the EQ-5D descriptive system and not the EQ-5D visual analogue scale. It should be noted that the values attached to the descriptive system reflect general population preferences, whilst the visual analogue scores are patient based. However, many decision-making bodies, such as the National Institute for Health and Clinical Excellence in England and Wales, highlight the importance of valuing health outcomes using population-based preferences of the type we have used for the broader comparative purposes of economic evaluation [2]. A fourth caveat to the study is that concerns about the empirical validity of EQ-5D and SF-6D utility scores should be counter-balanced by a rounded assessment of all psychometric properties of the measures in the maternity context. Although empirical validity is considered to provide the acid test for validity, other forms of validity, such as content validity, face validity, construct validity and valuation validity also require consideration by analysts. Moreover, other psychometric properties, such as practicality and reliability, are also of relevance.

## Conclusion

In conclusion, this study provides evidence that the SF-6D is an empirically valid and efficient alternative multi-attribute utility measure to the EQ-5D, and is capable of discriminating between external indicators of maternal health. Further research, which examines the psychometric properties of the EQ-5D, SF-6D and other multi-attribute utility measures in the maternity context, would strengthen the limited evidence base currently available to analysts conducting and interpreting economic evaluations.

## Competing interests

The authors declare that they have no competing interests.

## Authors' contributions

SP designed this empirical investigation and took the primary role in analysing the data and drafting the paper. JM and HS were the principal clinical investigators for the original postnatal support workers trial and contributed to iterative drafts of the paper.

## Supplementary Material

Additional file 1 Appendices A and B. Click here for file
