# Supplementary material for: Assessing the empirical validity of alternative multi-attribute utility measures in the maternity context
Source: Health Qual Life Outcomes. 2009 May 6;7:40. doi: 10.1186/1477-7525-7-40 (PMC2687423; doi:10.1186/1477-7525-7-40)
Supplement: Additional file 1 — Appendices A and B. [file 1477-7525-7-40-S1.doc]

**Appendix A: EQ-5D measure**

Please indicate which statement describes your own health state **today.** Please

answer all by placing a tick in **one** of the three options for each question.

**Mobility**

I have no problems in walking about

I have some problems in walking about

I am confined to bed

**Self-care**

I have no problems with self care

I have some problems with washing or dressing myself

I am unable to wash and dress myself

**Usual activities**

I have no problem in performing my usual activities (e.g. work, study, housework, leisure activity)

I have some problems in performing my usual activities

I am unable to perform my usual activities

**Pain/Discomfort**

I have no pain or discomfort

I have moderate pain or discomfort

I have extreme pain or discomfort

**Anxiety/Depression**

I am not anxious or depressed

I am moderately anxious or depressed

I am extremely anxious or depressed

To help people say how good or bad a health state is, we have drawn a scale (rather

like a thermometer) on which the best state you can imagine is marked by 100 and

the worst state is marked by 0.

We would like you to indicate on this scale how good or bad your own health is, in your

opinion. Please do this by drawing a **single line** from the box below to whichever point on the scale indicates how your health state is.

(BEST imaginable health state) **100**

**90**

**80**

**70**

Your own

health today

**60**

**50**

**40**

**30**

**20**

**10**

(WORST imaginable health state)

**0**

**Appendix B: SF-36 measure**

The following questions ask for your views about your health and how you feel about life in general. If you are unsure about how to answer any question, try and think about your overall health and give the best answer you can. Do not spend too much time answering, as your immediate response is likely to be the most accurate.

**1.** In general, would you say your health is: *(Please tick one box)*

| Excellent | | |  | Very good | | |  | Good | | |  | Fair | | |  | Poor | | |
| --- | --- | --- | --- | --- | --- | --- | --- | --- | --- | --- | --- | --- | --- | --- | --- | --- | --- | --- |
|  | | |  |  | | |  |  | | |  |  | | |  |  | | |
|  |  |  | | |  |  | | |  |  | | |  |  | | |  |  |

**2.** *Compared to one year ago*, how would you rate your health in general now? *(Please tick one box)*

| Much better now than one year ago | | |  | Somewhat better now than one year ago | | |  | About the same | | |  | Somewhat worse now than one year ago | | |  | Much worse now than one year ago | | |
| --- | --- | --- | --- | --- | --- | --- | --- | --- | --- | --- | --- | --- | --- | --- | --- | --- | --- | --- |
|  | | |  |  | | |  |  | | |  |  | | |  |  | | |
|  |  |  | | |  |  | | |  |  | | |  |  | | |  |  |

1. The following questions are about activities you might do during a typical day. Does your health now limit you in these activities? If so, how much? *(Please tick* ***one*** *box on each line)*

|  |  | **Yes,**  **Limited**  **a lot** |  | **Yes,**  **Limited a little** |  | **No, not**  **limited at all** |
| --- | --- | --- | --- | --- | --- | --- |

| a) | **Vigorous activities**, such as running, lifting heavy objects, participating in strenuous sports |  |  |  |  |  |
| --- | --- | --- | --- | --- | --- | --- |
|  |  |  |
|  |  |  |  |  |  |  |
| b) | **Moderate activities**, such as moving a table, pushing a vacuum, bowling or playing golf |  |  |  |  |  |
|  |  |  |
|  |  |  |  |  |  |  |
| c) | Lifting or carrying groceries |  |  |  |  |  |
|  |  |  |  |  |  |  |
| d) | Climbing **several** flights of stairs |  |  |  |  |  |
|  |  |  |  |  |  |  |
| e) | Climbing **one** flight of stairs |  |  |  |  |  |
|  |  |  |  |  |  |  |
| f) | Bending, kneeling or stooping |  |  |  |  |  |
|  |  |  |  |  |  |  |
| g) | Walking **more than a mile** |  |  |  |  |  |
|  |  |  |  |  |  |  |
| h) | Walking **half a mile** |  |  |  |  |  |
|  |  |  |  |  |  |  |
| i) | Walking **100 yards** |  |  |  |  |  |
|  |  |  |  |  |  |  |
| j) | Bathing and dressing yourself |  |  |  |  |  |

4. During the *past 4 weeks*, how much time have you had any of the following problems with your work or other regular daily activities *as a result of your physical health?* *(Please tick* ***one*** *box on each line)*

|  |  | **All of the time** |  | **Most of the time** |  | **Some of the time** |  | **A little of the time** |  | **None of the time** |
| --- | --- | --- | --- | --- | --- | --- | --- | --- | --- | --- |

| a) | Cut down on the **amount of time** you spent on work or other activities |  |  |  | |  |  |  |  |  |
| --- | --- | --- | --- | --- | --- | --- | --- | --- | --- | --- |
|  |  |  |  |  |  |  |  |  |
|  |  |  |  |  |  |  |  |  |  |  |
| b) | **Accomplished less** than you would like |  |  |  |  |  |  |  |  |  |
|  |  |  |  |  |  |  |  |  |  |  |
| c) | Were limited in the **kind** of work or other activities |  |  |  |  |  |  |  |  |  |
|  |  |  |  |  |
|  |  |  |  |  |  |  |  |  |  |  |
| d) | Had **difficulty** performing the work or other activities (eg it took more effort) |  |  |  |  |  |  |  |  |  |
|  |  |  |  |  |

5. During the **past 4 weeks**, how much time have you had any of the following problems with your work or other regular daily activities **as a result of any emotional problems** (such as feeling depressed or anxious)? *(Please tick* ***one*** *box on each line)*

|  |  | **All of the time** |  | **Most of the time** |  | **Some of the time** |  | **A little of the time** |  | **None of the time** |
| --- | --- | --- | --- | --- | --- | --- | --- | --- | --- | --- |

| a) | Cut down on the **amount of time** you spent on work or other activities |  |  |  | |  |  |  |  |  |
| --- | --- | --- | --- | --- | --- | --- | --- | --- | --- | --- |
|  |  |  |  |  |  |  |  |  |
|  |  |  |  |  |  |  |  |  |  |  |
| b) | **Accomplished less** than you would like |  |  |  |  |  |  |  |  |  |
|  |  |  |  |  |  |  |  |  |  |  |
| c) | Didn’t do work or other activities as **carefully** as usual |  |  |  |  |  |  |  |  |  |
|  |  |  |  |  |

6. During the **past 4 weeks**, to what extent have your physical health or emotional problems interfered with your normal social activities with family, friends, neighbours or groups? *(Please tick* ***one*** *box)*

| Not at all |  | Slightly |  | Moderately |  | Quite a bit |  | Extremely |
| --- | --- | --- | --- | --- | --- | --- | --- | --- |
|  |  |  |  |  |  |  |  |  |

|  |  |  |  |  |  |  |  |  |
| --- | --- | --- | --- | --- | --- | --- | --- | --- |

7. How much **bodily pain** have you had during the **past 4 weeks**?

| None |  | Very mild |  | Mild |  | Moderate |  | Severe |  | Very severe |
| --- | --- | --- | --- | --- | --- | --- | --- | --- | --- | --- |
|  |  |  |  |  |  |  |  |  |  |  |

|  |  |  |  |  |  |  |  |  |  |  |
| --- | --- | --- | --- | --- | --- | --- | --- | --- | --- | --- |

During the *past 4 weeks* how much did *pain* interfere with your normal work (including work both outside the home and housework)? *(Please tick* ***one*** *box)*

| Not at all |  | A little bit |  | Moderately |  | Quite a bit |  | Extremely |
| --- | --- | --- | --- | --- | --- | --- | --- | --- |
|  |  |  |  |  |  |  |  |  |

|  |  |  |  |  |  |  |  |  |
| --- | --- | --- | --- | --- | --- | --- | --- | --- |

*9. These questions are about how you feel and how things have been with you during the past 4 weeks. For each question please give the one answer that comes closest to the way you have been feeling. (Please tick* ***one*** box on each line)

|  | How much time during  **the last month**: | **All of the time** |  | **Most of the time** |  | **A good**  **bit of the time** |  | **Some of the time** |  | **A little of the time** |  | **None of the time** |
| --- | --- | --- | --- | --- | --- | --- | --- | --- | --- | --- | --- | --- |

| a) | Did you feel full of life? |  |  |  |  |  |  |  |  |  |  |  |
| --- | --- | --- | --- | --- | --- | --- | --- | --- | --- | --- | --- | --- |
|  |  |  |  |  |  |  |  |  |  |  |  |  |
| b) | Have you been very nervous? |  |  |  |  |  |  |  |  |  |  |  |
|  |  |  |  |  |  |  |  |  |  |  |  |  |
| c) | Have you felt so down in the dumps that nothing could cheer you up? |  |  |  |  |  |  |  |  |  |  |  |
|  |  |  |  |  |  |
|  |  |  |  |  |  |  |  |  |  |  |  |  |
| d) | Have you felt calm and peaceful? |  |  |  |  |  |  |  |  |  |  |  |
|  |  |  |  |  |  |  |  |  |  |  |  |  |
| e) | Did you have a lot of energy? |  |  |  |  |  |  |  |  |  |  |  |
|  |  |  |  |  |  |  |  |  |  |  |  |  |
| f) | Have you felt downhearted and low? |  |  |  |  |  |  |  |  |  |  |  |
|  |  |  |  |  |  |  |  |  |  |  |  |  |
| g) | Did you feel worn out? |  |  |  |  |  |  |  |  |  |  |  |
|  |  |  |  |  |  |  |  |  |  |  |  |  |
| h) | Have you been a happy person? |  |  |  |  |  |  |  |  |  |  |  |
|  |  |  |  |  |  |  |  |  |  |  |  |  |
| i) | Did you feel tired? |  |  |  |  |  |  |  |  |  |  |  |

10. During the *past 4 weeks*, how much of the time has your *physical health or emotional problems* interfered with your social activities (like visiting friends, relatives etc.)? *(Please tick* ***one*** *box)*

| All of the time |  | Most of the time |  | Some of the time |  | A little of the time |  | None of the time |
| --- | --- | --- | --- | --- | --- | --- | --- | --- |
|  |  |  |  |  |  |  |  |  |

|  |  |  |  |  |  |  |  |  |
| --- | --- | --- | --- | --- | --- | --- | --- | --- |

11. How TRUE or FALSE is *each* of the following statements for you? (*Please tick one box on each line)*

|  |  | **Definitely true** |  | **Mostly true** |  | **Not sure** |  | **Mostly**  **false** |  | **Definitely**  **false** |
| --- | --- | --- | --- | --- | --- | --- | --- | --- | --- | --- |

| a) | I seem to get ill more easily than other people |  |  |  |  |  |  |  |  |  |
| --- | --- | --- | --- | --- | --- | --- | --- | --- | --- | --- |
|  |  |  |  |  |
|  |  |  |  |  |  |  |  |  |  |  |
| b) | I am as healthy as anybody I know |  |  |  |  |  |  |  |  |  |
|  |  |  |  |  |  |  |  |  |  |  |
| c) | I expect my health to get worse |  |  |  |  |  |  |  |  |  |
|  |  |  |  |  |  |  |  |  |  |  |
| d) | My health is excellent |  |  |  |  |  |  |  |  |  |
